# The role of growth hormone in assisted reproduction

**DOI:** 10.3389/fendo.2022.1055097

**Published:** 2022-12-02

**Authors:** Alexander M. Quaas, Alan S. Penzias, Eli Y. Adashi

**Affiliations:** ^1^ Division of Reproductive Medicine and Gynecological Endocrinology (RME), University Hospital, University of Basel, Basel, Switzerland; ^2^ Boston IVF, Waltham, MA, United States; ^3^ Division of Reproductive Endocrinology and Infertility, Department of Obstetrics and Gynecology, Beth Israel Deaconess Medical Center, Boston, MA, United States; ^4^ Obstetrics, Gynecology, and Reproductive Biology, Harvard Medical School, Boston, MA, United States; ^5^ Department of Medical Science, Warren Alpert Medical School, Brown University, Providence, RI, United States

**Keywords:** growth hormone, Assisted Reproductive Technology, adjuvants in IVF, poor responders, add-ons

## Abstract

In contemporary ART, the use of “add-ons” during ovarian stimulation has increased, especially in poor responders. Growth Hormone (GH) is an adjunctive therapy that has been studied extensively in the translational and clinical setting, with an ongoing scientific debate over its effectiveness and optimal use. In this review, we aim to provide an overview of the physiologic basis for the use of GH in ART, and to summarize the latest evidence regarding its clinical use, primarily as an adjunct to ovarian stimulation, but also in the IVF lab and with regards to its effects on the endometrium.

## Introduction

The indication for In-Vitro Fertilization (IVF) resulting in the first successful birth *via* Assisted Reproductive Technology (ART) was tubal factor infertility (1). Since then the indications for IVF have expanded to cover virtually all of the pathologies causing infertility, including male factor, ovulation disorders and decreased ovarian reserve. The latter, often occurring in the setting of advanced reproductive age, has proven to be the biggest challenge to ART success, with no clear effective remedies available to counteract age-related fertility decay (2).

The definition of “poor responder” has differed widely in the literature, and various criteria for inclusion in this group have been proposed. The parameters used isolated or in combination to define the poor ovarian response patient include female age, various ovarian reserve markers, and previous intermediate IVF cycle outcomes such as history of cycle cancellation, serum estradiol concentration on day of trigger or number of oocytes retrieved (3).

More commonly used “poor responder” classification systems include the Bologna criteria (4) and the POSEIDON criteria (5).

For “poor responders” to ovarian stimulation, numerous adjuvants or “add-ons” have been used in an attempt to increase live birth rates (6). Growth Hormone (GH) is one of the adjuvants that have received significant attention, and a heated scientific debate exists regarding its effectiveness.

In this review, we aim to explore the physiologic basis for the use of GH in ART, and then discuss the role of GH as an adjunct for ovarian stimulation, in the IVF laboratory, and with regard to the endometrium before outlining ethical aspects regarding the use of GH in ART.

## The physiologic basis for the use of growth hormone in ART

Numerous translational studies point to an important role of GH in the setting of ovarian stimulation.

GH is a polypeptide produced and secreted by the anterior pituitary gland, primarily at night during sleep. Given its length of 191 amino acids, it theoretically meets the size criteria for classification as a protein but lacks the tertiary and quaternary chain structure (7). Although GH has direct actions, most of its effects are mediated *via* Insulin-Like Growth Factors (IGFs), predominantly IGF-1, and include skeletal, visceral and general body growth. GH receptors (GHR) are expressed in the liver, adipose and muscle tissues, but also in ovarian granulosa cells and endometrial epithelial cells (8, 9), and a permissive role for the somatotrophic axis in the reproductive process has long been suspected (10). Abundant expression of GHR messenger RNA (mRNA) and GHR immunoreactivity were shown in large ovarian antral follicles, but not in preantral follicles of goat GCs (11). GH and its intermediary IGF-1 promote granulosa cell (GC) function and proliferation (12). In cell culture experiments, IGF-1 reduces apoptosis in granulosa-lutein cells in a dose-dependent fashion (13).IGF-1 increases the action of FSH on its receptor, augmenting estradiol secretion by GCs (14). It has been demonstrated in several female mammals that IGFs appear to stimulate the growth of ovarian follicles just before their entry into the terminal growth phase, which is highly dependent on FSH (15). In heifers, recombinant bovine somatotropin (BST) was shown to increase the population of antral follicles in a 1991 study by Gong et al. (16) This effect did not appear to be mediated through changes in circulating gonadotropin concentrations or gonadotropin receptor levels, but *via* increased peripheral IGF-l concentrations or even a direct effect of BST at the level of the ovary. Receptor binding sites for IGF-1 and GH have been described and localized in different compartments of the sow ovary, including in granulosa and theca cells of healthy follicles (17).

In humans, the GHR density in GCs increases with age in normal, but not poor responders according to a 2018 translational study by Regan et al. (18). In this study, GH treatment in women aged 39-45 with an antral follicle count (AFC) of ≤ 8 increased the density of GHR, but also the density of follicle-stimulating hormone receptor (FSHR), luteinizing hormone/choriogonadotropin receptor (LHCGR) and bone morphogenetic protein receptor type-1B (BMPR1B).

## Growth hormone as an adjunct for ovarian stimulation

GH has been used as an adjunct to ovarian stimulation for several decades, mostly for “poor responders”. A major challenge to research into the efficacy of GH in this setting has been the heterogeneity of GH treatment doses and regimens, and the inconsistent definition of “poor responders”. However, several randomized controlled trials (RCTs) on the use of GH as adjuvant medication during ovarian stimulation have been published and their data synthesized in meta-analyses.

Tesarik et al.conducted one such RCT in 100 patients aged over 40 years, randomized to GH co-treatment versus placebo (19).

The two groups were similar with regards to the mean age (42.2 versus 42.3), mean basal follicle-stimulating hormone (FSH; 10.2 versus 10.1 IU/ml) and the number of prior IVF attempts (2.9 versus 2.8).

GH was given at a dose of 8 IU daily from stimulation day 7 until the day of egg retrieval. A similar number of oocytes and embryos were obtained in both groups, however the clinical pregnancy rates (26% versus 6%) and live birth rates (22% versus 4%) were statistically significantly superior in the GH arm.

At least four meta-analyses have been conducted on the topic of GH co-treatment during ovarian stimulation in poor ovarian responders (20–23).

The first one, a 2003 Cochrane analysis including 3 RCTs, reported an increase in live birth rates with GH adjuvant therapy (OR 4.37, 95% CI 1.06 to 18.01) but the sample size was low and the 95% CI bordered on 1, indicating that the finding was “only just significant” (20). The second meta-analysis, in 2009 by Kolibianakis et al., included 169 “poor responders from 6 RCTs (21). The risk difference for the live birth rate (LBR) across the 6 included RCTs was 0.17 (95% confidence interval 0.05-0.3) in favor of the addition of GH. In addition, there was a lower cycle cancellation rate in the GH arm. The third meta-analysis from 2017 by Li et al. included 565 poor responders from 11 RCTs for the primary outcome of live birth rate (22). The clinical pregnancy rate and live birth rate were significantly increased in the GH arm. There was also an increase in the intermediate cycle parameters estradiol on day of trigger and number of mature (MII) oocytes in favor of GH use. Additionally, across the included RCTs, GH use was associated with decreases in the rate of cycle cancellation and the total gonadotropin dose used.

These findings have led investigators to postulate in recent years that co-treatment with GH in poor responders may be cost-effective and to advocate increased utilization of GH in this setting (24).

However, controversy still exists over the use of GH based on more recent evidence. A double-blind, placebo-controlled, RCT from Australia in women with a previously documented poor response to FSH stimulation reported live birth rates of 9/62 (14.5%) for women co-treated with GH during IVF and 7/51 (13.7%) for the placebo group (risk difference 0.8%, 95% confidence interval [CI] –12.1 to 13.7%; odds ratio [OR] 1.07, 95% CI 0.37–3.10) (25). The authors observed greater odds of undergoing an egg retrieval in the GH-treated women, but no better chance of embryo transfer or live birth.

In keeping with this finding, the fourth and most recent systematic review and meta-analysis on this topic by Cozzolino et al. from 2020 cast doubt on a beneficial effect of GH on live birth rates in poor responders (23). It included 12 RCTs of poor ovarian responders undergoing a single IVF/ICSI cycle with GH supplementation versus conventional controlled ovarian stimulation, with the primary outcome of live birth rate, and secondary outcomes of clinical pregnancy rate (CPR), miscarriage rate, ongoing pregnancy rate (OPR), number of oocytes, number of mature (metaphase II [MII]) oocytes and the number of embryos available for transfer (23). Between the 586 women assigned to the intervention and the 553 assigned to the control group, there was no significant difference in live birth rate (risk ratio 1.34, 95% CI 0.88-2.05), miscarriage rate or ongoing pregnancy rate. GH supplementation was associated with an increased CPR, number of oocytes retrieved (mean difference 1.62), number of MII oocytes (mean difference 2.06), and number of embryos available to transfer (mean difference 0.76) (23). The authors concluded that GH supplementation in poor responders may improve some reproductive outcomes, but not the most crucial outcome of live birth rates.

In patients who are not classified as poor responders by the Bologna criteria (4), a recent retrospective cohort study of 41 women by Skillern et al. evaluated whether daily cotreatment with GH could improve IVF outcomes in patients with a lower than expected number of MII oocytes, poor blastulation rate, and/or lower than expected number of euploid embryos for their age in their first cycle of IVF/PGT-A (26). The total number of biopsied blastocysts (mean ± SD; 2.0 ± 1.6 vs 3.5 ± 3.2, p = 0.009) and euploid embryos (0.8 ± 1.0 vs

2.0 ± 2.8, p = 0.004) were significantly increased in the adjuvant GH cycle compared to the control cycle. However, the retrospective nature of the study with patients serving as their own controls made it susceptible to significant bias *via* regression to the mean, and live birth rates were not reported (27).

Further research is necessary to clarify the clinical efficacy of GH administration, especially with regards to the most relevant endpoint of live birth rates.

## Growth hormone use in the IVF laboratory

In the ART laboratory, GH has demonstrated promising preliminary results when added to culture media in various settings.

*In vitro* maturation (IVM) of immature oocytes has been a challenging endeavor and an active area of scientific investigation over the last few decades. Rates of successful IVM with the addition to the culture media for immature oocytes were reported in various animal models (28–31). In 2019, Li et al. demonstrated that GH promotes IVM of human oocytes (32).

They collected human germinal vesicle (GV) oocytes, cultured them with different GH concentrations, and assessed rates of successful maturation to the MII stage. In GV oocytes cultured without GH, the maturation rate was 35%, whereas maturation rates to the MII stage ranged between 50 and 70% for GV oocytes cultured in media with GH concentrations between 10 and 1000 ng/ml. The highest maturation rate was observed at a concentration of 200 ng/ml.

**Figure 1:**
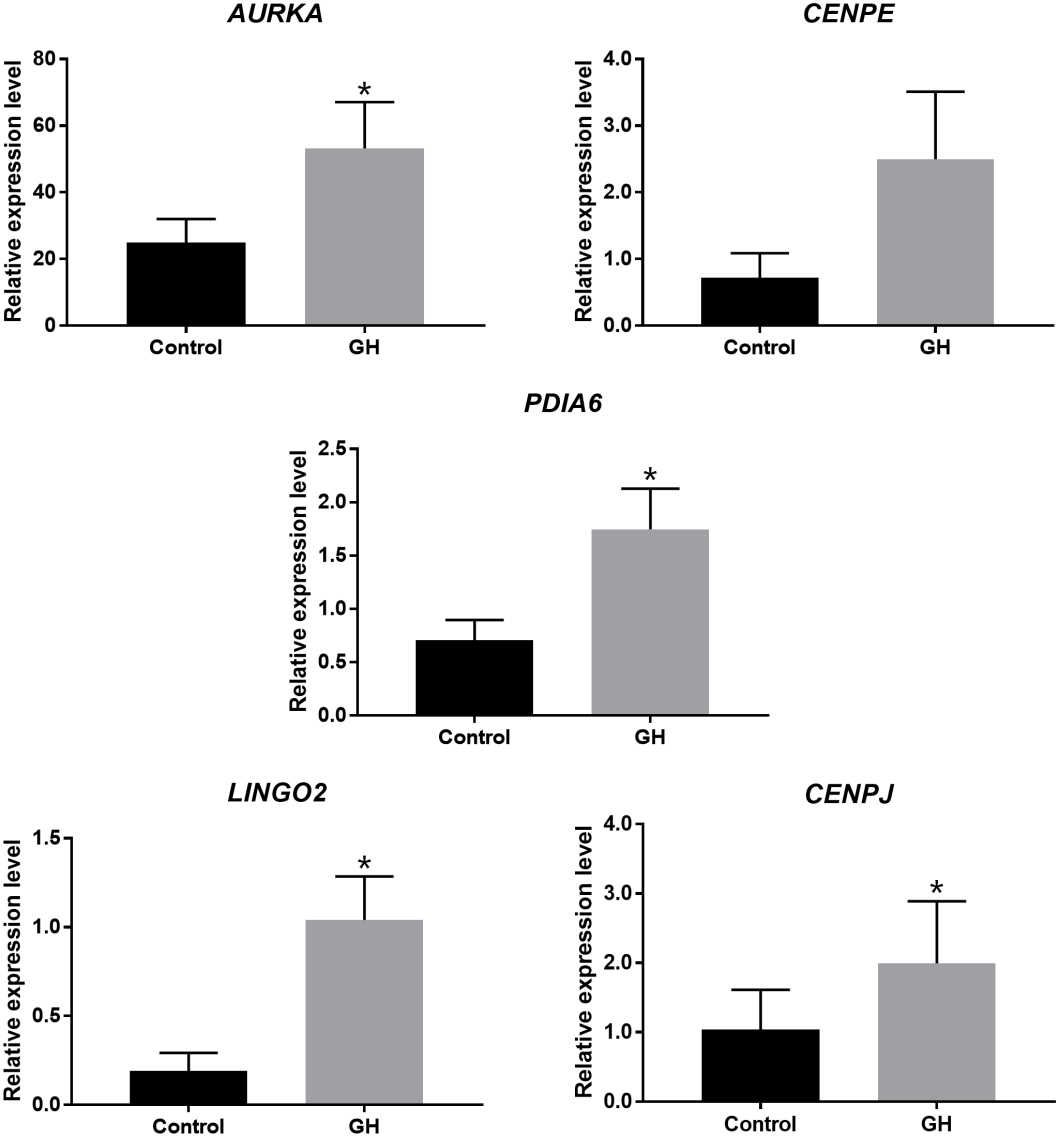
Expression of genes associated with meiotic progression and embryo development in human oocytes cultured with and without GH, assessed by Real-time PCR *P < 0.05 [From (32)].

The investigators found increased fertilization and blastocyst formation rates at this GH concentration compared to control oocytes, however this finding did not reach statistical significance. They conducted gene expression analyses using single-cell RNA-sequencing and real-time PCR and reported enhanced expression of genes associated with meiotic progression and embryo development, including AURKA, PDIA6, LINGO2 and CENPJ (**Figure 1**, (3The foundation for live birth in IVF is successful implantation, which requires a competent embryo, a receptive endometrium, and an adequate interplay between the two to achieve embryo-endometrial synchrony. There is an emerging body of evidence to suggest that GH has a positive effect on endometrial receptivity (9).

**Figure 2:**
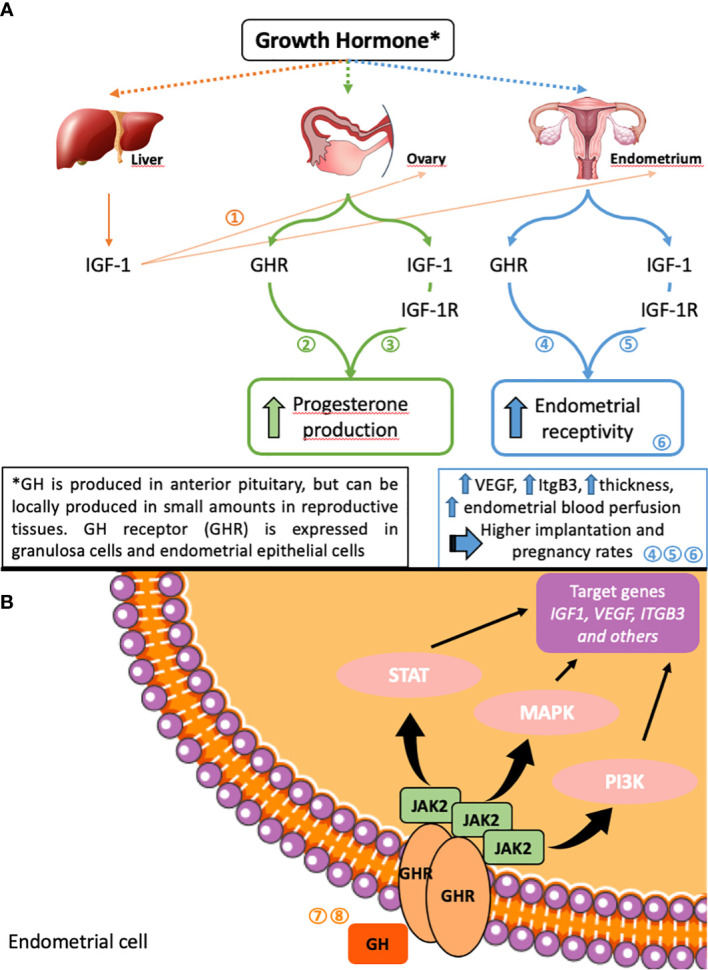
Possible mechanisms of GH effects on ovarian and endometrial function **(A)** and on endometrial cells **(B)**. Adapted from (9).

Basic science research on the endometrial carcinoma cell line RL95-2 demonstrated that the administration of GH significantly up-regulated the expression IGF-1 and the receptivity-related factors VEGF and ITGB3 in endometrial cells at both the mRNA and protein level (33). GH expression has been demonstrated in endometrial biopsies in the secretory, but not proliferative phase (34) and GH is produced locally in endometrial cells in small amounts in addition to systemic production by the anterior pituitary gland [**Figure 2** (9)]. Locally and systemically produced GH is thought to act in synergy to promote endometrial receptivity (35). Furthermore, evidence from animal studies suggests that GH may exert an indirect effect on the endometrium by aiding to maintain the corpus luteum (36). Direct intrauterine perfusion of GH has been shown to promote regeneration of thin rat endometria in a pilot animal model (37).

Clinical studies have shown promising results for GH co-treatment in ART patients with recurrent implantation failure and/or a history of thin endometrium with regard to improvements in endometrial thickness and implantation rates in fresh and frozen IVF cycles (9). It is feasible that improvements in IVF outcomes with GH co-treatment are only partially due to an effect on the oocyte and embryo quality, but also due to a beneficial effect on endometrial receptivity. Further investigation is necessary to elucidate this hypothesis.

## Ethical aspects regarding the use of growth hormone in assisted reproduction

The use of add-ons in ART is controversial (38). Patients desiring to become parents are presented with an increasingly large menu of adjunct treatment options. This is especially the case for the most vulnerable patient populations, such as those with a poor ovarian reserve and advanced reproductive age and those who suffer from recurrent pregnancy loss or recurrent implantation failure. Often treatments are recommended prior to demonstration of benefit with regards to the most important outcome, live birth, and with minimal regulation (39).

These adjunct therapies increase the complexity of the IVF process and the overall cost of treatment (40).

A recent review scrutinizing the benefit of commonly used add-ons concluded that little high-quality evidence for most adjunct therapies existed, and that “large, well-designed, randomized trials must be conducted to evaluate the effectiveness and safety of these interventions” (40). The authors of this 2019 review evaluated the available evidence for 10 commonly used add-on interventions in contemporary clinical IVF practice, rated the quality of the evidence, and then assigned a color code to the summary of evidence. Green indicated “high-quality evidence showing effectiveness”; amber “small body of evidence or conflicting evidence which means further research required and technique not recommended for routine use”; and red “no evidence to show it is effective and safe”. No evaluated adjunct therapy was assigned a green color code and almost half (4 of 10) received a red label of “no evidence to show it is effective and safe”.

For GH, the authors deemed the quality of evidence to be “very low” or “not available” and assigned the amber color code to the use of GH as an adjunct in poor responder populations.

The principles of medical ethics, first published by Beauchamp and Childress in 1979, include autonomy, beneficence, non-maleficence and justice (41). According to the principles of beneficence and non-maleficence, medical interventions with little or no proven benefit should be avoided. Should our- often well informed- patients have the autonomy to request adjunct treatments when the evidence is inconclusive?

Furthermore, is it always in the best interest of the patient to await “large, well-designed, randomized trials” for every aspect of ART prior to implementation?

Our field abounds with examples of interventions that were implemented without a rigorous assessment of safety and benefit. It is doubtful whether an institutional review board (IRB) or ethics committee would approve a plan for an RCT on the use of intracytoplasmic sperm injection (ICSI) in humans if the technique had not already been invented three decades ago (42).

From the active debate regarding the use of GH as an adjunct therapy in ART it is evident that individual interpretation of the available evidence varies greatly.

It appears that neither a blanket recommendation for GH use in ART nor a complete denial of its effectiveness are consistent with the “scientific truth”. Detailed patient counseling is necessary when using GH in contemporary clinical practice.

## Conclusion and future directions

Growth Hormone (GH) is a promising therapy in the field of ART, with solid biologic plausibility for its use. GH may be of benefit as an adjunct therapy in ovarian stimulation, especially for poor responders. Its use has also been studied in the IVF laboratory and with regards to its effects on the endometrium. More well-designed research is needed to explore the optimal use of GH in ART to allow for optimal counseling and treatment of patients.

## Author contributions

Each author participated actively and sufficiently in this review, and all authors made substantial contributions to its concept, and design. All authors contributed to the article and approved the submitted version.

## Funding

RME Basel Research Fund; Boston IVF Research Fund.

## Conflict of interest

The authors declare that the research was conducted in the absence of any commercial or financial relationships that could be construed as a potential conflict of interest.

## Publisher’s note

All claims expressed in this article are solely those of the authors and do not necessarily represent those of their affiliated organizations, or those of the publisher, the editors and the reviewers. Any product that may be evaluated in this article, or claim that may be made by its manufacturer, is not guaranteed or endorsed by the publisher.
